# VGLUT2 Functions as a Differential Marker for Hippocampal Output Neurons

**DOI:** 10.3389/fncel.2018.00337

**Published:** 2018-10-02

**Authors:** Christian Wozny, Prateep Beed, Noam Nitzan, Yona Pössnecker, Benjamin R. Rost, Dietmar Schmitz

**Affiliations:** ^1^Strathclyde Institute of Pharmacy and Biomedical Sciences, University of Strathclyde, Glasgow, United Kingdom; ^2^Neuroscience Research Center, Charité – Universitätsmedizin Berlin, Berlin, Germany; ^3^Berlin Institute of Health, Berlin, Germany; ^4^German Center for Neurodegenerative Diseases (DZNE), Berlin, Germany; ^5^NeuroCure – Cluster of Excellence, Charité – Universitätsmedizin Berlin, Berlin, Germany; ^6^Einstein Center for Neurosciences, Berlin, Germany

**Keywords:** channelrhodopsin-2, entorhinal cortex, hippocampus, subiculum, synaptic transmission, VGLUT2

## Abstract

The subiculum is the gatekeeper between the hippocampus and cortical areas. Yet, the lack of a pyramidal cell-specific marker gene has made the analysis of the subicular area very difficult. Here we report that the vesicular-glutamate transporter 2 (VGLUT2) functions as a specific marker gene for subicular burst-firing neurons, and demonstrate that VGLUT2-Cre mice allow for Channelrhodopsin-2 (ChR2)-assisted connectivity analysis.

## Introduction

The hippocampal formation consists of anatomically defined brain areas such as the dentate gyrus (DG), the cornu ammonis (area CA1-CA3 in rodents), the subiculum (SUB), pre- and parasubiculum, and the entorhinal cortices (EC), all of which fulfill a variety of different tasks. Well documented is the role of these hippocampal subregions in spatial navigation.

The SUB is the main hippocampal output structure and functions as a relay between the hippocampus proper and the EC. Two different types of subicular pyramidal neurons have been described based on their intrinsic firing pattern as burst- or regular-firing neurons (Stewart and Wong, 1993; Taube, 1993; Staff et al., 2000). These neurons project to different brain regions: burst-firing neurons to the medial EC, the presubiculum, the retrosplenial cortex, and to the hypothalamus, whereas mostly regular-firing neurons project to the amygdala, the lateral EC and the nucleus accumbens (Kim and Spruston, 2012). Remarkably, CA1 inputs to these two cell-types express different forms of synaptic plasticity (Fidzinski et al., 2008; Wozny et al., 2008; Aoto et al., 2013). The intrinsic firing pattern, however, is not static, but might be modulated by neuronal activity (Moore et al., 2009). Until now, the lack of cell-specific marker genes for burst- and regular-firing neurons has, however, hampered the application of state-of-art circuit analysis tools such as Channelrhodopsin-2 (ChR2), which would allow to redefine the cortico-hippocampal wiring diagram and to further disentangle the role of these particular neurons.

## Materials and Methods

### Ethics Statement and Animal Handling

Animal husbandry and experimental procedures were performed in accordance with the guidelines of local authorities (Berlin, Germany), the German Animal Welfare Act, and the European Council Directive 86/609/EEC. Animals were housed on a 12:12 h day-night cycle with food and water available *ad libitum*.

### Molecular Biology and Adeno-Associated Virus Delivery to Transgenic Mice

Two different Cre-dependent vector were used, firstly, an AAV-CAG-DIO-hChR2(H134R)-mCherry/ or -EYFP vector, and secondly, a Switch vector. The Cre-dependent Switch vector for AAVs was created as following: The WPRE element in pAAV-EF1α-double floxed-hChR2(H134R)-EYFP-WPRE-HGHpA (Addgene 20298) was deleted by ClaI digestion and relegation, in order to increase the packaging capacity of the vector. The 5′ Lox2272 and LoxP sites were removed by a SalI/AscI digestion, and Lox2272 and LoxP sites flanking a SV40 nuclear localization-sequence (NLS, encoding PKKKRKV) were inserted by oligo-annealing. The coding sequence of mRuby2 was PCR amplified from Addgene clone 50943 and inserted downstream of the NLS, resulting in pAAV-EF1α-Switch:NLSmRuby2/ChR2(H134R)-EYFP-HGHpA. This vector enables expression of NLS-mRuby2 in Cre-negative cells, and of ChR2(H134R)-EYFP in Cre-positive neurons. Constructs were packaged into AAV serotypes 1 and 9 using published protocols (Rost et al., 2015).

Viral particles (200 to 500 nl) were injected in the SUB of VGLUT2-ires-Cre mice (Slc17a6^*tm*2(*cre*)*Lowl*^ knock-in (Vong et al., 2011) aged three to 5 weeks. In these mice Cre recombinase expression is tightly coupled to VGLUT2 expression, and can be found in glutamatergic neurons in various brain region. The coordinates for viral injections were adjusted depending on the age of the animal and the bregma-lambda length: For a P31 animal, for example, the following coordinates were used: AP ± 2.66, ML ± 3.79, and DV -2.8.

### Electrophysiology

Horizontal slices were prepared at 2 to 3 weeks after the injections using a sucrose-based artificial cerebrospinal fluid containing 87 mM NaCl, 50 mM sucrose, 26 mM NaHCO_3_, 2.5 mM KCl, 1.25 mM NaH_2_PO_4_, 0.5 mM CaCl_2_, 7.0 mM MgCl_2_, and 25 mM glucose, saturated with 95% O_2_ and 5% CO_2_ (pH 7.4). Recordings were performed at room temperature (22–25°C) in artificial cerebrospinal fluid containing (in mM): 125 NaCl, 25 NaHCO_3_, 10 glucose, 3 KCl, 2 CaCl_2_, 1 MgCl_2_, 1.25 NaH_2_PO4.

Drugs were applied to block synaptic transmission at the following concentrations: AMPA receptor antagonist NBQX, 25 μM; NMDA receptor antagonist D-APV, 50 μM; GABA_A_ receptor antagonist gabazine, 1–2 μM; GABA_B_ receptor antagonist CGP55845, 10 μM. For circuit mapping the following drugs were used: TTX, 1 μM; and 4-AP, 100 μM. All drugs are purchased from Tocris, Bio-Techne GmbH, Wiesbaden, Germany, with the exception of 4-AP (Sigma, Sigma-Aldrich Chemie GmbH, München, Germany).

The intracellular solution contained (in mM): 135 potassium-gluconate, 6 KCl, 2 MgCl_2_, 0.2 EGTA, 5 Na_2_-phosphocreatine, 2 Na_2_-ATP, 0.5 Na_2_-GTP, 10 4-(2-hydroxyethyl)-1-piperazineethanesulfonic acid (HEPES) buffer, and 0.2% biocytin. The pH was adjusted to 7.2 with potassium hydroxide (KOH).

### Data Acquisition

Recordings were performed using Multiclamp 700A/B amplifiers (Molecular Devices). Data sampled at 5–20 kHz and filtered at 2–10 kHz was acquired using either Igor (Wavemetrics) or pClamp (Molecular Devices).

### Light Stimulation

Excitation light from a mercury lamp (in conjunction with a TTL-controlled mechanical shutter from Uniblitz, Vincent Associates, NY, United States) was applied via an Olympus 60x objective to activate ChR2. The light intensity was measured to 2.7 mW/mm^2^.

### Histology

Slices were fixed overnight, washed with phosphate buffered saline (PBS), processed, and mounted on a slide using previously published protocols (Wozny and Williams, 2011). Confocal images were acquired using a Leica SP5 confocal microscope.

### Statistical Analysis

Excel (Microsoft) or GraphPad Prism (GraphPad Software) were used for statistical analysis. No power calculations were performed to determine sample sizes prior to the study, but similar cohorts were used in a previous study (Rost et al., 2015). Data were compared with a Mann-Whitney test, and displayed as mean ± standard error of the mean (s.e.m.).

## Results

To identify promising candidate genes for SUB-specific expression pattern we screened public repositories such as the Allen Brain Atlas. A differential search between area CA1 of the hippocampus and the SUB further aided to narrow the number of genes, and revealed VGLUT2 as one of the most promising candidates (**Supplementary Figure S1**).

**FIGURE 1:**
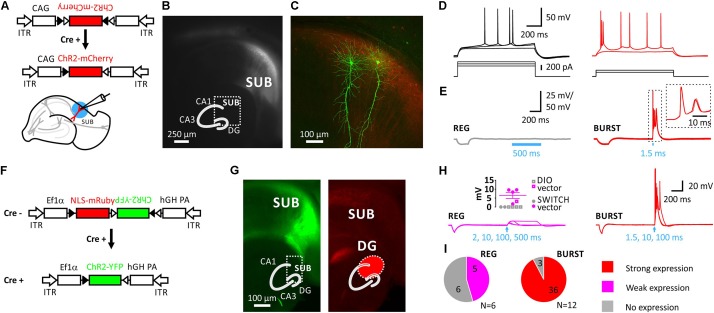
Selective expression of Cre-dependent ChR2 in subicular burst-firing neurons in VGLUT2-Cre mice. **(A)**, Injection of AAVs into the subiculum (SUB) of VGLUT2-Cre mice. Schematic showing the Cre-conditional DIO-ChR2-mCherry construct and the slice preparation. Blue circle illustrates blue light illumination. **(B)**, Live image of mCherry fluorescence showing mCherry-positive neurons in the SUB. Inset shows schematic of hippocampus with DG, CA3, CA1, and SUB. **(C)**, Maximum projection of confocal z-stacks of biocytin-filled neurons and virally induced expression of the red fluorophore. **(D)**, Firing pattern of a subicular regular-firing (REG, left), and of a subicular burst-firing neuron (BURST, right). **(E)**, Left: Average of 10 traces using a 500 ms light pulse demonstrated no expression of ChR2 in REG neuron. Right: In contrast, a 1.5 ms light pulse elicited action-potential firing in subicular BURST neuron; inset shows 10 consecutive traces. Recordings were performed in the presence of blockers of synaptic transmission. **(F)**, Switch vector design allowing NLS-mRuby expression in Cre-negative and ChR2-YFP expression in Cre-positive neurons. **(G)**, Left: EYFP signal is restricted to SUB, whereas NLS-mRuby is seen in the distal CA1 region, SUB and the DG (on the right). Infected area indicated in red in inset. **(H)**, Left: Blue-light stimulation of up to 500 ms (indicated by arrow) elicited no or small depolarizations of <10 mV due to no or weak expression of ChR2 in regular-firing (REG) neurons. Inset shows evoked depolarization (*n* = 5; *N* = 3 mice). Closed circles indicate mice infected with Switch vector, color code as in **(I)**. Right: Strong expression of ChR2 resulted in action-potential firing in BURST neurons (*n* = 36 out of 39 neurons; *N* = 12 mice). **(I)**, None of the REG neurons showed strong expression of ChR2, which means none fired APs (*n* = 11 neurons; *N* = 6 mice), in contrast to 36 out of 39 BURST neurons showing light-induced AP-firing (*N* = 12 mice). Data pooled from both Cre-conditional constructs.

Injection of an adeno-associated virus (AAV) encoding Cre-dependent DIO (double-floxed inverted orientation) ChR2(H134R)-mCherry into the SUB of VGLUT2-Cre mice resulted in localized expression of ChR2-mCherry (**Figures 1A–C**). To test the functional expression of ChR2 we recorded light-evoked responses in the presence of synaptic blockers (**Figures 1D,E**).

We first classified SUB neurons in respect to their intrinsic firing pattern into burst- and regular firing neurons (**Figure 1C**). Very little difference was found between both cell types with respect to their intrinsic electrophysiological properties in response to hyper- or depolarizing pulses except for the initial firing frequency, which actually defines burst-firing (**Supplementary Figure S2**).

However, expression of ChR2 nicely coincided with the intrinsic firing pattern as subsequent light stimulation elicited action potentials (AP) only in intrinsically burst-firing neurons (**Figures 1E,H,I**). Post-hoc confocal imaging of the biocytin-labeled recorded neurons confirmed the typical characteristics of pyramidal neurons (**Figure 1C**), as well as co-localization of the biocytin signal and the virally induced expression of ChR2-mCherry (**Supplementary Figure S3**).

We next created a Cre-dependent Switch vector that allowed us to identify both Cre-positive neurons by ChR2-YFP expression, as well as Cre-negative neurons by a red fluorophore (**Figure 1F**). In Cre-negative cells, the Ef1α promoter drives expression of mRuby2 fused to a nuclear-localization sequence (NLS), whereas the ChR2-YFP coding sequence is reversely orientated. Cre-mediated recombination of the construct removes the NLS-mRuby2 coding sequence and initiates ChR2-YFP expression by inverting the ChR2-YFP sequence. This technique allowed us to identify the infected brain area more precisely, and also to distinguish infected from uninfected neurons (**Figure 1G**).

Regarding the expression of ChR2 in subicular neurons the response patterns upon blue light illumination were further analyzed and grouped into three categories: (i) strong, (ii) weak, and (iii) no response. A strong response was considered to be an AP. In 36 out of 39 burst-firing neurons light elicited an AP (AP size: 108.7 ± 1.8 mV, *n* = 36; **Figure 1H**). A weak response was smaller than 10 mV following either a 10, 100, 200, or 500 ms light pulse. Five out of eleven regular-firing neurons showed such a response (6.5 ± 1.7 mV, range 1.7–9.6 mV, *n* = 5; see inset **Figure 1H**). No expression of ChR2, and therefore no response to light was evident in 3 out 39 burst-firing (8%), and in 6 out of 11 regular-firing neurons (55%; **Figure 1I**).

Previously, subicular neurons have been shown to project to the deep layers of the entorhinal cortex (Kloosterman et al., 2003). More recently the wiring has been refined demonstrating that subicular (and CA1) pyramidal neurons project onto Coup-TF interacting protein 2 (CTIP2)-expressing neurons located in layer 5b of the EC (Surmeli et al., 2016). However, whether these subicular neurons express VGLUT2 is unclear.

**FIGURE 2:**
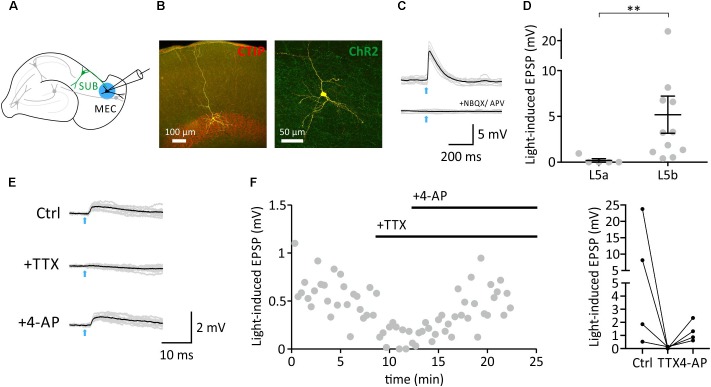
Monosynaptic connections from the SUB to mEC L5b neurons. **(A)**, Schematic showing ChR2-infected burst-firing neuron in the SUB, and subsequent whole-cell recordings from mEC neuron. **(B)**, Anatomical reconstruction of an EC L5b pyramidal neuron. Yellow: Biocytin-streptavidin-Alexa647 signal. Red: Immunoreactivity against CTIP. Green: ChR2-YFP. **(C,D)**, Light-induced postsynaptic responses (EPSPs; illumination indicated by an arrow, 5 ms) of mEC L5a (*n* = 5, *N* = 4 mice) and L5b pyramidal neurons (**D**, *n* = 11, *N* = 6 mice, Mann-Whitney test, *p* < 0.01). **(E)**, Light stimulation (indicated by an arrow, 5 ms) elicited an EPSP (left), which was blocked by TTX, and rescued with 4-AP confirming monosynaptic nature of the signal. **(F)**, Left, time course. Right, individual 4-AP experiments (*n* = 4, *N* = 3 mice).

We therefore recorded from layer 5b neurons in the EC and applied ChR2-assisted mapping circuit in VGLUT2-Cre mice to monitor efferent monosynaptic connections of subicular burst-firing neurons (**Figures 2A–D**). Subicular efferents were stimulated using blue light pulses (2–5 ms; **Figure 2C**). Application of either glutamate receptor blockers (**Figure 2C**), or the sodium channel blocker tetrodotoxin (TTX; **Figures 2E,F**) abolished the response, confirming that the synaptic response was driven by AP-triggered transmitter release. Addition of the potassium channel blocker 4-aminopyridine (4-AP) rescued the light-evoked EPSP (**Figures 2E,F**), proving the monosynaptic nature of the synaptic input. In contrast, and consistent with previous reports (Surmeli et al., 2016), layer 5A neurons did not receive inputs from subicular burst-firing neurons (**Figure 2D**; Mann-Whitney test, *p* < 0.01).

## Discussion

Here we report the identification of VGLUT2 as a marker of subicular burst-firing neurons. By utilizing VGLUT2-Cre mice in combination with viral gene delivery strategies of Cre-dependent ChR2-expression constructs we demonstrate that VGLUT2 expression is mainly found in subicular burst-firing neurons. This versatile tool allows microcircuit analysis confirming and extending previous results of where hippocampal output neurons synapse onto mEC L5b neurons (Tamamaki and Nojyo, 1995; Naber et al., 2001; Kloosterman et al., 2003; Surmeli et al., 2016).

Recently, the restricted expression of fibronectin-1 (FN1) in the dorsal subiculum was utilized to generate a mouse line expressing Cre specifically in dorsal SUB neurons. Subsequently, optogenetic manipulations were performed to address the role of the dorsal SUB in memory formation (Roy et al., 2017). Another recent study divided proximal and distal subicular pyramidal neurons (Cembrowski et al., 2018). Proximal subicular neurons express neuronatin (Nnat), whereas neurotensin (Nts) is found in distal neurons. However, it is currently not known whether these marker genes specifically label subpopulations of subicular principal neurons.

Földy et al. (2016) performed a single-cell transcriptome study of subicular pyramidal neurons. Following electrophysiological characterization the mRNA of burst- and regular-firing neurons was collected, and sequenced. There was no difference in the number of detected genes between these two types of subicular neurons, and only a small number of exclusively expressed genes were found, however, none of these were further validated as a functional genetic marker. We used a differential approach. Viruses were used to infect Cre- and non-Cre-recombinase expressing neurons. In our hands, over 90% of the recorded burst-firing neurons expressed high amounts of ChR2, whereas none of the subicular regular-firing neurons expressed sufficient amounts of ChR2 to drive AP firing following blue light illumination with varying lengths.

Of note, the expression of VGLUT1 and VGLUT2 in the brain is thought to be complementary: VGLUT1 is mainly expressed in cortical areas, whereas VGLUT2 is expressed in subcortical areas such as the thalamus, amygdala or hypothalamus (Hisano et al., 2000; Fremeau et al., 2001; Herzog et al., 2001). A few brain areas including the SUB, however, seem to express both, VGLUT1 and VGLUT2 (Ishihara and Fukuda, 2016; Kinnavane et al., 2018). Whether VGLUT1-positive neurons in the SUB are mainly of the regular-firing type has to be determined, as has the role of both types of subicular neurons during behavior.

## Author Contributions

CW and DS designed the experiments. CW, PB, NN, and YP performed research. CW, PB, NN and BRR analyzed data. BRR generated molecular tools. CW wrote the paper with help of PB, BRR, and DS. All authors read and edited the final version of the manuscript.

## Conflict of Interest Statement

The authors declare that the research was conducted in the absence of any commercial or financial relationships that could be construed as a potential conflict of interest.
