# Supplementary material for: VGLUT2 Functions as a Differential Marker for Hippocampal Output Neurons
Source: Front Cell Neurosci. 2018 Oct 2;12:337. doi: 10.3389/fncel.2018.00337 (PMC6176088; doi:10.3389/fncel.2018.00337)
Supplement: Supplementary file 1 [file Data_Sheet_1.PDF]

SUPPLEMENTARY INFORMATION

VGLUT2 functions as a differential marker for hippocampal output neurons

Christian Wozny[1,2,7,*], Prateep Beed[2,3,7], Noam Nitzan[2], Yona Pössnecker[2], Benjamin R. Rost[2,4], and Dietmar Schmitz[2,4,5,6]

[1]Strathclyde Institute of Pharmacy and Biomedical Sciences, University of Strathclyde, 161 Cathedral Street, Glasgow, G4 0RE, UK

[2]Charité – Universitätsmedizin Berlin, Neuroscience Research Center, Charitéplatz 1, 10117 Berlin, Germany

[3]Berlin Institute of Health, Anna-Louisa-Karsch-Str. 2, 10178 Berlin, Germany

[4]DZNE – German Center for Neurodegenerative Diseases, Charité – Universitätsmedizin, Charitéplatz 1, 10117 Berlin, Germany

[5]NeuroCure – Cluster of Excellence, Charité – Universitätsmedizin Berlin, 10117 Berlin, Germany

[6]Einstein Center for Neurosciences, 10117 Berlin, Germany

[7]These authors contribute equally to this study.

*For Correspondence: christian.wozny@strath.ac.uk

Supplementary Figure 1: Gene expression

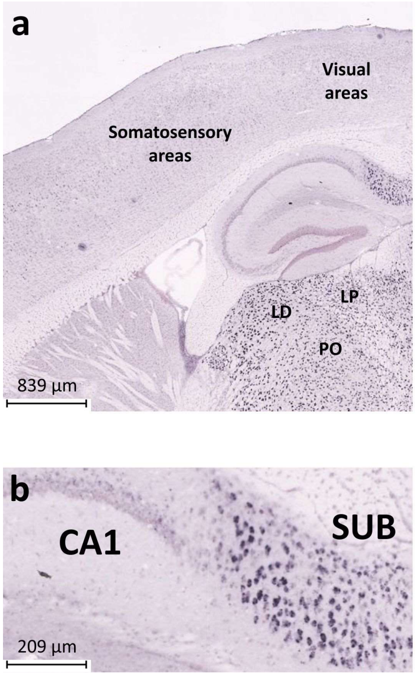

Gene expression of Gene Slc17a6 (solute carrier family 17 (sodium-dependent inorganic phosphate cotransporter), member 6, alias VGLUT2 – vesicular glutamate transporter). In-situ hybridization modified from the Allen Brain Atlas repository (http://mouse.brain-map.org/gene/show/80230). Sagittal slice.

a, No expression of VGLUT2 in the visual and the somatosensory areas.
b, Enlargement of **a**. Strong expression in the subiculum (SUB), but no labeled neurons in area CA1 of the hippocampus. Abbreviations: LD: Lateral dorsal nucleus of the thalamus; LP: Lateral dorsal nucleus of the thalamus (LP); PO: Posterior complex of the thalamus.

Supplementary Figure S2: Intrinsic electrophysiological properties of subicular burst- (BURST) and regular-firing (REG) cells

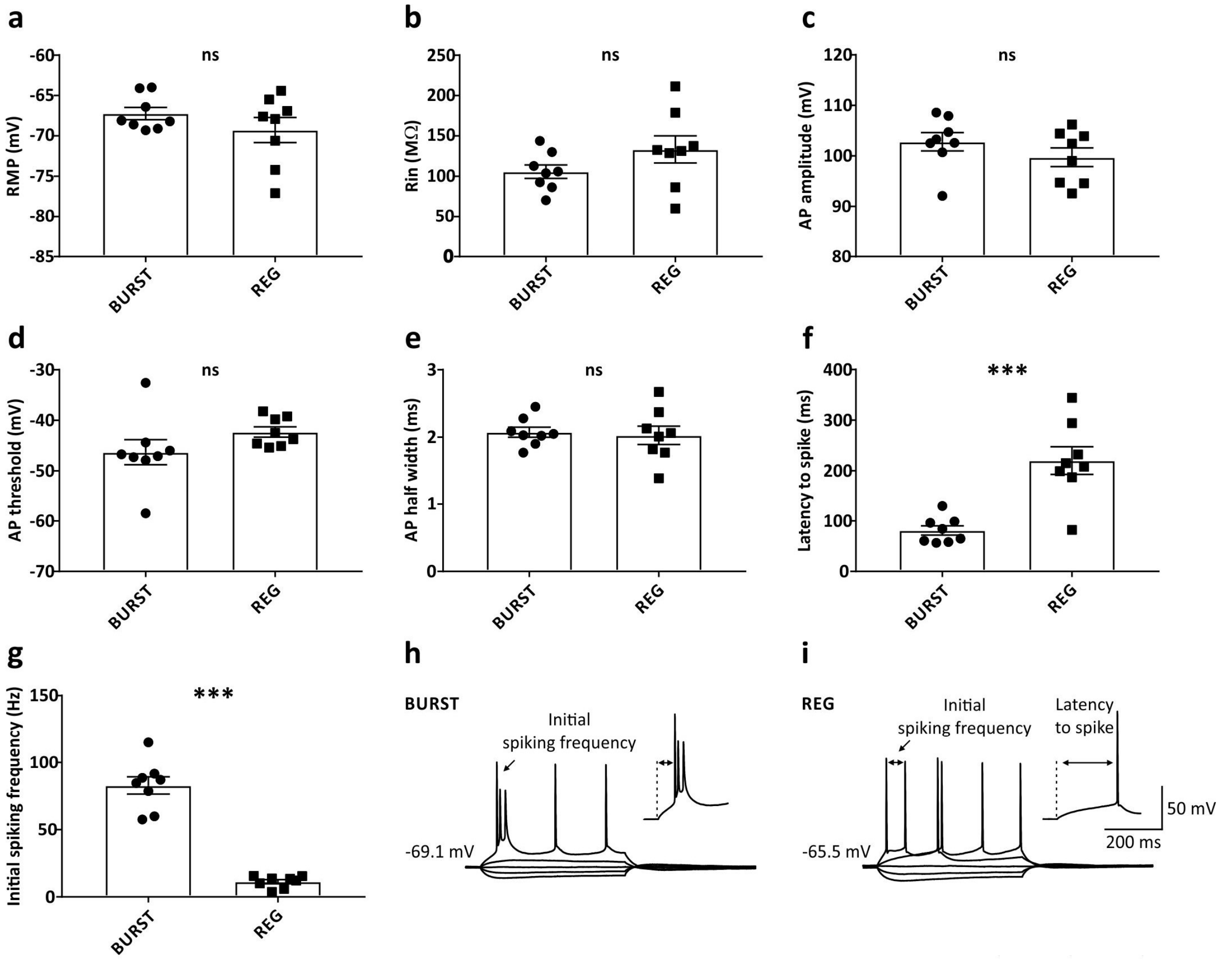

a - **g**, Eight randomly selected neurons were analysed for both cell types (BURST and REG). **h** and **i**, Example traces; current injected in 40 pA steps from -80 pA to 80 pA.

Supplementary Figure S3: Single confocal plan

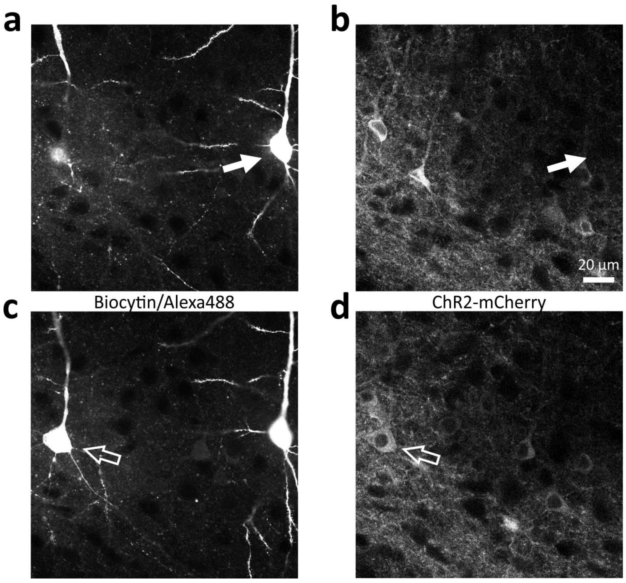

a, Single confocal plan (1 µm) of two biocytin-filled subicular pyramidal neurons (Alexa 488). **b**, Single confocal plan of ChR2-mCherry-labelled infected neurons. Please note that the biocytin-filled neuron is negative for mCherry (regular firing neuron; closed arrow). **c**, Subicular burst-firing cell, which is positive for mCherry (open arrow; **d**).
